# Effects of ultrasound-assisted Maillard reaction on the emulsifying and flavor properties of brewer’s spent grain protein–gum arabic conjugates

**DOI:** 10.1016/j.ultsonch.2026.107755

**Published:** 2026-01-29

**Authors:** Mingyu Kim, Hyunwoo Ahn, Yujin Wang, Beomkyung Cho, Woojin Na, Kwang-Geun Lee

**Affiliations:** Department of Food Science and Biotechnology, Dongguk University-Seoul, 32, Dongguk-ro, Ilsandong-gu, Goyang-si, Gyeonggi-do 10326, the Republic of Korea

**Keywords:** Brewer’s spent grain protein, Gum arabic, Maillard reaction, Emulsifying properties, Volatile compounds

## Abstract

This study explores the effects of ultrasound-assisted heating compared to conventional wet heating on the Maillard reaction (MR) between brewer’s spent grain (BSG) protein and gum arabic (GA), focusing on various factors like reaction kinetics, structural changes, emulsifying performance, and volatile formation. Ultrasound process markedly accelerated glycation, with a 45 min sonicated sample glycation to a similar degree to that induced by traditional 3 h treatment, such that cavitation facilitates early MR pathways. Both approaches enhanced solubility and remodeled secondary structures, although in different manners: conventional heating promoted a gradual increase in β-sheet content and a decrease in α-helix content, while ultrasound induced rapid unfolding and structural reorganization. All MRPs enhanced the emulsifying properties, but the conventional sample after 3 h obtained the highest emulsifying activity index (EAI) and emulsion stability index (ESI) values. Samples treated with ultrasound showed moderate improvement, however, at considerably reduced reaction times. Dynamic light scattering (DLS) and fluorescence microscopy have also confirmed that conventionally heated MRPs form smaller and more uniform droplets. Volatile profiling indicated that conventional heating produced a broader spectrum of aldehydes, ketones, and furans associated with off-flavors, whereas ultrasound greatly reduced compounds such as (E,E)-2,4-decadienal and 2-pentylfuran, indicating a flavor-protective potential. In general, the reaction efficiencies and flavor quality of ultrasound-assisted heating are higher, and emulsifying performance is optimized by the conventional heating technology. This work shows that BSG protein–GA conjugates are versatile enough to achieve clean-label, plant-based emulsifier applications and its desired functional/sensory properties.

## Graphical abstract

**Figure:**
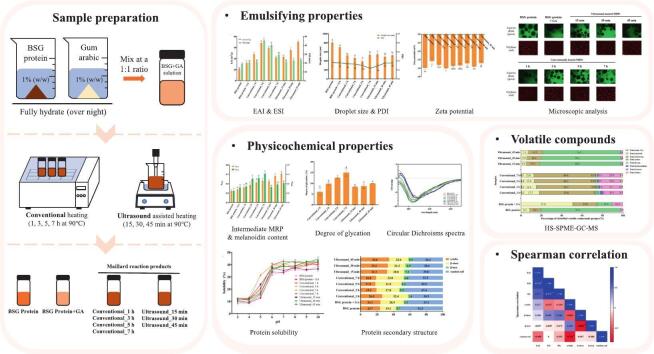

## 1 Introduction

Beer, a beverage consumed for centuries, remains popular to date. During the beer production process, barley is sprouted to produce malt, which undergoes mashing and lautering processes. These processes yield a solid byproduct known as brewer’s spent grain (BSG). It is estimated that the production of 1 m^3^ of beer generates approximately 200 kg of BSG. Given that global beer production exceeds 1.75 × 10^11^ L annually, a substantial quantity of BSG is produced. The disposal of BSG disposal imposes significant costs and may lead to adverse environmental effects. BSG has notably high protein content, ranging from 15 to 30% (w/w) [1]. Consequently, ongoing research efforts have focused on protein extraction from BSG and the enhancement of its properties. Techniques such as enzymatic hydrolysis and sonication have been investigated to enhance protein functionality [2]. By employing advanced methods to upcycle BSG, it is possible to reduce disposal costs while creating a valuable source of protein.

The Maillard reaction (MR), also known as non-enzymatic browning, is a chemical process that occurs in three stages: the initial, intermediate, and final stages. During the initial stage, the carbonyl group of carbohydrates reacts with the amino group of proteins to form protein-carbohydrate conjugates. These conjugates exhibit superior emulsifying properties [3]. Numerous studies have investigated the Maillard reaction [4], [5]. The two primary methods employed for the MR between proteins and polysaccharides are dry heating and wet heating. Notably, the wet-heating method has a shorter reaction time compared to the dry heating method [6]. Furthermore, combining the wet-heating method with ultrasound has been shown to further enhance the emulsifying properties [7]. Gum arabic (GA), used as the carbon source in this study, possesses excellent water solubility and low solution viscosity. GA’s high solubility facilitates uniform dispersion and promotes chemical reactions, effectively mitigating the limitations of BSG protein, such as its low solubility. Additionally, GA’s low viscosity ensures that the continuous phase of the emulsion does not become excessively thick, enabling a clearer assessment of the emulsifying properties induced by the MR on proteins [8]. BSG proteins are characterized by abundant hydrophobic residues and repetitive amino acid sequences, mainly derived from prolamin (hordein) and glutelin fractions [9]. They are also rich in glutamic acid and proline, which contribute to a relatively flexible molecular conformation. These structural and compositional features provide intrinsic advantages during the Maillard reaction by promoting efficient protein–polysaccharide conjugation [10].

An emulsion is a colloidal system where two or more immiscible phases, with one phase dispersed as droplets within another [11]. The mixing of two immiscible phases creates a thermodynamically unstable state, necessitating the use of an emulsifier to reduce surface tension and stabilize the dispersed droplets in the continuous phase [12]. Artificial emulsifiers are widely utilized in industrial applications. However, recent research suggests that commonly used artificial emulsifiers, such as carboxymethylcellulose (CMC) and polysorbate-80 (P80), may trigger minor inflammatory responses in the host [13]. This has driven increasing consumer demand for natural, clean-label emulsifiers [14]. Proteins, as natural biomolecules with amphiphilic properties, can serve as natural emulsifiers. BSG offers a promising protein source for this purpose. The primary challenge in utilizing BSG protein for emulsification is the high hydrophobicity of its main component, prolamine [1]. Glycation with highly hydrophilic GA can enhance the emulsifying properties of BSG protein, expanding its potential applications.

This study aimed to induce a reaction between BSG protein and GA through conventional wet heating and ultrasound-assisted wet heating, followed by an investigation of the resulting physicochemical, emulsifying, and flavor properties. Although numerous studies have focused on improving the solubility of BSG protein, the enhancement of its water solubility and emulsifying ability through Maillard reactions with polysaccharides remain uninvestigated. To assess the emulsifying properties, the emulsion activity index (EAI) and emulsion stability index (ESI) were determined, followed by an analysis of droplet size and distribution characteristics. A comprehensive set of experiments was conducted to evaluate the physicochemical properties, including protein solubility, Maillard reaction products, degree of glycation, and circular dichroism analyses. Volatile compounds were identified using headspace solid-phase microextraction (HS-SPME), followed by gas chromatography-mass spectrometry (GC–MS). This study provides valuable insights into the development of plant-based emulsifiers and the effective utilization of BSG protein.

## 2 Materials and methods

### 2.1 Chemical reagents and materials

BSG used in this study was produced at the Jeonju Brewery of Hitejinro Co., Ltd. (Seoul, Republic of Korea). The BSG contained 77.76% (w/w) moisture and 6.48% (w/w) protein. It was stored at −80℃ in a deep freezer until use. A C7-C40 n-alkane standard was obtained from Supelco, Inc. (Bellefonte, PA, USA). Canola oil was purchased from Sajo Haepyo Co., Ltd. (Seoul, Korea) at a local food market in Goyang-si, Gyeonggi-do, Korea. Other reagents, including 2-octanol, Nile Red, Coomassie Brilliant Blue R-250, sodium dodecyl sulfate (SDS), fluorescein 5(6)-isothiocyanate (FITC), β-mercaptoethanol, glycerol, and gum arabic, were procured from Sigma-Aldrich Chemical Co. (St. Louis, MO, USA).

### 2.2 Extraction of BSG protein

The frozen BSG was thawed at room temperature (25℃). The wet BSG was then dried using a food dehydrator (LD-9118, L’EQUIP, Korea) at 50℃ for 24 h or until a constant weight was achieved. A total of 100 g of dehydrated BSG was weighed and finely ground using a blender (SMKANB-4000, Poongnyun Co., Ltd., Korea). The resulting BSG powder was sieved through a 355 µm sieve and stored in an airtight container with a silica gel desiccant bag at room temperature (25℃). The extraction method for BSG protein was slightly modified from the procedure described by Li, Yang, Coldea and Zhao [15]. The procedure was adapted as follows: BSG powder was combined with a 110 mM NaOH solution at a 1:20 (w/v) ratio. A 500 mL slurry was prepared and stirred at 300 rpm using a magnetic mixer (MSH-20D, Daihan Scientific Company Ltd., Korea) for 2 h to extract the BSG protein. The slurry was then centrifuged at 10℃ and 8000*g* for 20 min. The supernatant was collected, and its pH was adjusted to 3.8 using a pH meter (Orion Star A211, Thermo Fisher Scientific, Waltham, MA, USA) and a 2 M HCl solution. The solution was centrifuged again under the same conditions, and the resulting supernatant was discarded to obtain a pellet. The pellet was washed twice with deionized water (D.W.) and subsequently neutralized to pH 7.0 using a 2 M NaOH solution. The neutralized solution was lyophilized in a freeze dryer (FD8508, Ilshin Biobase, Korea) at −78℃ for 3 days. The lyophilized BSG protein powder was ground using a grinder (Mini Grinder, Ankric, China) and stored in a specimen cup with a silica gel desiccant bag at room temperature (25℃). The crude protein concentration of the resulting BSG protein was determined using the Kjeldahl method [16], yielding 56.7 ± 3.1% (w/w).

### 2.3 Preparation of Maillard Reaction Products (MRPs)

MRPs were prepared using BSG protein and GA following the methods described by previous studies [17] with slight modifications. The BSG protein and GA were individually dispersed in 0.2 M phosphate buffer (pH 7.2) to give a final concentration of 1% (w/w), and the mixtures were stirred overnight to ensure complete solubilization. The BSG protein solution was combined with the GA mixture at a 1:1 (v/v) ratio. Based on the results of preliminary experiments conducted under various temperature conditions (data not shown), the 90 °C heating condition was selected as the final condition for preparing the MRPs sample. To prepare conventional MRPs, 20 mL aliquots of the BSG protein + GA mixture were transferred into 20 mL screw-cap vials, sealed with Teflon tape, and heated in a shaking water bath (MSH-20D, Daihan Scientific Company Ltd., Korea) at 90 °C and 150 rpm for 1, 3, 5, and 7 h. After heating, the vials were immediately transferred to an ice bath to terminate the reaction. For ultrasound-assisted MRPs, 40 mL of the BSG protein + GA mixture was placed in a beaker, which was then placed in a larger beaker containing hot water on a temperature-controlled magnetic stirrer to maintain 90 °C. The ultrasonic probe (VCX130, probe diameter 0.6 cm, Output power 130 W and a frequency of 20 kHz, Sonics & Material, Inc., Newtown, USA) was immersed 0.2 cm into the solution, and sonicated at an amplitude level of 80% in pulse mode (2 s on/2 s off), delivering a total energy input of 45–136 kJ, for 15, 30, or 45 min. The actual power density of sonication was measured at 44.5 ± 0.4 W/cm^2^ and calculated as following Eq. (1):

(1) $$\mathrm{Power}\;density\;\left(W/{\mathrm{cm}}^{2}\right)=\frac{J/time\;(sec)}{\mathrm{Surface}\;area\;of\;probe\;tip\;\left({\mathrm{cm}}^{2}\right)}$$

### 2.4 Physicochemical properties of MRPs

#### 2.4.1 Intermediate MRPs and melanoidin content

The intermediate MRPs and melanoidin content were analyzed spectrophotometrically using the method described by Habinshuti, Zhang, Sun and Mu [18] with slight modifications. A solution was prepared by mixing 0.75 mL of undiluted MRP solution with 14.25 mL of a 0.1% SDS solution, resulting in a final solid fraction of 5% (w/v). The solution was vortexed for 3 s. A UV–Vis spectrophotometer (Optizen™ Alpha, K LAB, Daejeon, Korea) was used to measure absorbance at 294 nm and 420 nm, with 0.1% SDS solution serving as the blank. The optical density values of the sample mixture at both wavelengths were recorded, and the blank values were subtracted from the corresponding absorbance values. Absorbance at 294 nm was used to estimate the levels of Maillard reaction intermediate products, and absorbance at 420 nm was used to estimate the level of melanoidins.

#### 2.4.2 Degree of Glycation (DG)

The degree of glycation (DG) was analyzed using a colorimetric method with o-phthaldialdehyde (OPA), following the method described by Jiang, Huangfu, Jiang, Wang, Bao and Ma [19]. To prepare the OPA reagent, 80 mg of OPA was dissolved in 2 mL of methanol. This solution was combined with 50 mL of 0.1 M sodium tetraborate buffer (pH 9.7), 5 mL of 20% (w/w) SDS solution, and 200 µL of β-mercaptoethanol. The volume was adjusted to 100 mL using D.W. A 200 µL aliquot of the MRP solution, containing 5 mg/mL of protein was blended with 4 mL of the OPA reagent and vortexed for 3 s. The reaction mixture was incubated for 2 min in a 35℃ water bath, and the absorbance was measured at 340 nm using a UV–Vis spectrophotometer. The DG (%) was calculated using the following Eq. (2):

(2) DG (%) = (A_pg_ – A_s_) × 100/Ap

where A_pg_ is the optical density of the protein-gum arabic mixture at 340 nm, A_s_ is the optical density of the sample at 340 nm, and A_p_ is the optical density of the protein solution at 340 nm.

#### 2.4.3 Protein solubility

To assess protein solubility, MRPs were diluted four-fold with D.W and thoroughly mixed. The pH of each sample was adjusted incrementally from 3 to 10 in steps of 1. Each sample was mixed continuously for 30 min. Subsequently, centrifugation was performed at 10,000*g* for 30 min at 20 °C. Samples were collected before centrifugation and the supernatant was collected after centrifugation. A bicinchoninic acid (BCA) assay was performed on each sample. The assay was conducted according to the microplate procedure outlined in the Pierce™ BCA Protein Assay Kit manual (Thermo Fisher Scientific, UK) using a 96-well cell culture plate (Cat. no. 31096, SPL Life Sciences Co., St. Petersburg, FL, USA), and absorbance was measured at 570 nm with a microplate reader (AMR-100, Hangzhou Allsheng Instruments Co., Ltd., China). Protein content was quantified by substituting each absorbance into a standard curve prepared using bovine serum albumin (BSA) provided in the kit. Protein solubility was calculated as the ratio of protein present in the supernatant to the total protein content in the solution. The formula for protein solubility is as following Eq. (3):

(3) Protein solubility (%) = (C_s_/C_t_) × 100

where C_s_ is the protein concentration in the supernatant, and C_t_ is the protein concentration in the solution.

#### 2.4.4 Protein secondary structures of MRPs

The secondary structural components of proteins, including α-helix, β-sheet, β-turn, and random coil, were investigated using a circular dichroism instrument (Chirascan, Applied Photophysics Ltd., Surrey, UK). The protein concentration of the supernatant, obtained after centrifugation of the sample at 1000*g* for 30 min, was determined and the sample was subsequently diluted to a final concentration of 0.25 mg/mL in 45 mM phosphate buffer. Analysis was conducted using a quartz glass cuvette with an optical length of 0.5 mm (106-QS, Hellma, Germany), into which the diluted samples were transferred. The scan range was set from 190 to 260 nm (time-per-point 0.25 s, 1 nm bandwidth). Prior to analysis, the instrument was purged with nitrogen at room temperature (25 °C). A 45 mM phosphate buffer-filled cuvette was used to obtain a background spectrum, with measurements performed in triplicate. The obtained CD spectra were analyzed using the BeStSel method (https://bestsel.elte.hu/index.php) to quantify the percentages of each secondary structural component: α-helix, β-sheet, β-turn, and random coil.

### 2.5 Emulsifying properties of MRPs

#### 2.5.1 Emulsion activity index (EAI) and emulsion stability index (ESI)

The emulsifying properties of the MRPs were evaluated by determining the EAI and ESI, following the method described by Pirestani, Nasirpour, Keramat, Desobry and Jasniewski [20]. To prepare the emulsion, 16 mL of the sample solution was combined with 4 mL of canola oil. The mixture was homogenized at 20,000 rpm for 2 min using a homogenizer. Immediately after homogenization, 50 μL of the emulsion was collected from the bottom of the container. After 10 min, another 50 μL sample was extracted from the same emulsion. Each sample was immediately diluted to 5 mL with 0.1% (w/v) SDS solution and vortexed for 3 s. The absorbance of the diluted emulsions was measured at 500 nm using a spectrophotometer, with 0.1% (w/v) SDS solution serving as a blank. All measurements were performed in triplicate. The EAI and ESI were calculated using Eqs. (4), (5):

(4) EAI (m^2^/g) = (2 × 2.303 × A_0_ × N)/(10000 × L × C × Φ)

(5) ESI (min) = (A_0_ × 10)/(A_0_ − A_10_)

where A_0_ is the absorbance at 0 min, A_10_ is the absorbance at 10 min, N is the dilution factor, L is the pathlength of the cuvette (cm), C is the protein concentration (g/mL), and Φ is the oil fraction.

#### 2.5.2 Dynamic light scattering (DLS) analysis

The emulsions were prepared by mixing 16 mL of a 0.5% (w/v) sample solution with 4 mL of canola oil, followed by homogenization at 20,000 rpm for 2 min using a homogenizer. Each emulsion was further homogenized in an ice bath using an ultrasound processor operating at an intensity of 77.0 ± 0.4 W/cm^2^ for 5 min to produce a nano-emulsion. The prepared emulsions were stored in an ice box until further analysis. Particle size, polydispersity index (PDI), and zeta potential were measured using a Zetasizer Nano ZS90 (Malvern Panalytical, UK) following the DLS analysis method described by Noh and Lee [11] For particle size and PDI measurements, samples were diluted ten-fold with D.W. After thorough dispersion, 1 mL of the diluted emulsion was transferred to a 10 mm optical path polystyrene disposable cuvette (DTS0012, Malvern Panalytical, UK). The refractive index of canola oil was set to 1.471, and the refractive index and dielectric constant of the dispersant (water) were set to 1.330 and 78.5, respectively. Measurements were performed in triplicate at 25℃ following a 30 s equilibration period, and the z-average and PDI values were recorded. For zeta potential measurements, the emulsions were diluted 100-fold with D.W. After ensuring thorough dispersion, the diluted samples were transferred to a folded capillary cell (DTS1070, Malvern Panalytical, UK). The oil and water parameters applied in the particle size and PDI measurements were used. Following a 30 s equilibration period at 25℃, the zeta potential was measured in triplicate.

#### 2.5.3 Inverted fluorescence microscopy

Each 0.5% (w/v) sample solution (10 mL) was mixed with 10 mL of canola oil and homogenized at 10,000 rpm for 4 min. The emulsion structure was analyzed based on the method described by Noh and Lee [11] with slight modifications. A 1 mL aliquot of the resulting emulsion was immediately stained with 4 µL of Nile Red (0.01 g/mL in 2-propanol) and 10 µL of FITC (0.02 g/mL in ethanol), with 1 min of vortexing following the addition of each stain. The stained emulsion was then diluted two-fold with a 0.1% SDS solution. A 40 µL aliquot was applied on a slide glass and covered with a cover slip. Images of the oil and aqueous phases were captured using an inverted fluorescence microscope (LS40, LEAM solution, Korea) with a 40 × objective lens.

### 2.6 Volatile compounds in MRPs

The volatile compound profile of MRPs was analyzed using headspace-solid phase microextraction (HS-SPME) coupled with gas chromatography-mass spectrometry (GC–MS). A 50/30 µm Divinylbenzene/Carboxen/Polydimethylsiloxane (DVB/CAR/PDMS) coated SPME fiber needle (57348-U, Supelco) was used for HS-SPME. First, 5 mL of the MRP sample (0.5 g protein/100 mL) was placed in a GC glass vial. Then, 10 µL of 2-octanol (diluted 50,000 times in ethyl alcohol) was added as an internal standard, followed by 1.5 g of NaCl to facilitate analyte volatilization. The vial was then sealed with a silicone/polytetrafluoroethylene (silicone/PTFE) magnetic cap (Gerstel, Germany).

The sealed vials were processed using a multipurpose sampler (Gerstel, Germany). Equilibration was conducted at 70℃ for 10 min with agitation at 250 rpm. Following equilibration, the fiber was exposed to the sample for 40 min at 70℃ for analyte absorption. The absorbed analytes were desorbed in the injection port of an Agilent 7820A GC system coupled with an Agilent 5977E mass spectrometry (MS) detector (Agilent Technologies, CA, USA) in splitless mode at 230℃ for 5 min. Separation was performed on a DB-WAX UI column (60 m × 25 mm × 0.5 µm; Agilent Technologies, CA, USA), using helium as the carrier gas at a flow rate of 1.0 mL/min. The oven temperature program was initiated at 44℃ for 5 min, followed by a ramp of 3℃/min to 170℃, held for 10 min. The temperature was then increased to 200℃ at a rate of 8℃/min and held for 10 min. A post-run temperature of 250℃ was maintained for 5 min. The inlet temperature was set to 250℃, and the interface temperature was set to 230℃. The MS detector was operated in full scan mode with electron impact (EI) ionization at 70 eV. The scan range was set from 30 to 350 *m*/*z*. Each sample was analyzed in triplicate.

Qualitative analysis was performed by comparing the mass spectra with the Wiley mass spectral library (NIST, Wiley), with a resemblance percentage above 85%, and calculating the Kovats retention index (RI). RI values were determined by analyzing a C7–C40 n-alkane standard mixture under the same chromatographic conditions and comparing the retention times. The relative quantification of the detected peaks was achieved by determining the ratio of each compound’s peak area to the area of the internal standard, 2-octanol.

### 2.7 Statistical analysis

All results, except for protein secondary structure, are expressed as mean ± standard deviation (S.D.) of triplicate measurements. One-way analysis of variance (ANOVA) was performed using IBM SPSS Statistics 23 (IBM, Chicago, USA) to determine significant differences (*p*-value < 0.05). Duncan’s multiple range test was then performed to identify groups with significant differences, which are indicated by different letters. Correlations between structural parameters and functional properties of BGS protein + GA MRPs were determined by Sperman rank test under SPSS software.

## 3 Results and discussion

### 3.1 Physicochemical properties of MRPs

#### 3.1.1 Degree of glycation (DG)

**Fig. 1:**
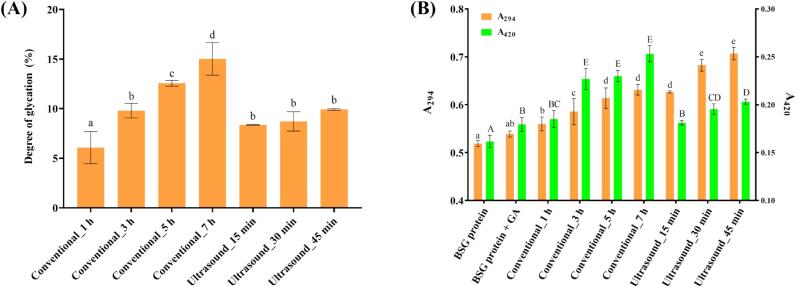
(A) Degree of glycation values of each MRPs. (B) Optical density of 294 nm (intermediate MRPs) and 420 nm (melanoidin).

The OPA reagent reacts with free amino groups in proteins to produce a fluorescent response. This allows for the use of the DG value as an index for quantifying the extent of amino group depletion compared to the control, reflecting the extent of the Maillard. In Fig. 1A, the DG value exhibited a time-dependent increase in conventionally heated samples. Similarly, samples subjected to ultrasound-assisted heating exhibited high DG values. The observed increase in DG values through conventional heating is consistent with findings of previous report [21]. During the Maillard reaction, the carbonyl moiety of the polysaccharide initially binds to exposed amino groups. Subsequently, with continued heating, additional binding occurs with newly exposed ε-amino groups in partially unfolded proteins, leading to an increase in DG. Ultrasound-assisted heating samples followed a trend consistent with previous studies, showing a more rapid increase in DG values compared to conventional heating. Notably, the ultrasound_45 min sample (9.9 ± 0.1) and the conventional_3 h sample (9.8 ± 0.7) exhibited similar DG values, despite the significantly shorter heating time for the ultrasound-treated sample. In the previous report, it attributed this trend to the energy provided by ultrasound treatment, which enhances the glycation process by physically bringing the reactants closer together [22]. Additionally, ultrasound can disrupt the quaternary structure of proteins, increasing the reactivity and efficiency of the reaction.

#### 3.1.2 Intermediate MRPs and melanoidin content

Fig. 1B presents the absorbance values of 294 nm and 420 nm in the samples. Absorbance at 294 nm corresponds to light-colored intermediate MRPs, including Amadori compounds, 5-(hydroxymethyl)furfuryl, while at 420 nm, absorbance indicates the presence of dark-colored final MRPs, such as melanoidins [23]. The observed increase in absorbance can be attributed to the formation of MRPs through the condensation reactions between the amino groups of proteins and the carbonyl groups of polysaccharides. Both conventional and ultrasound-assisted samples showed increases in absorbance at 294 nm and 420 nm with heating time. Ultrasound-assisted heated samples showed much higher absorbance at 294 nm and 420 nm after a short heating time than conventional-heated samples. In particular, the ultrasound-assisted sample exhibited higher absorbance at the early stages of MRPs, forming in a short time due to cavitation-induced local hotspots, enhanced mass transfer, and temporary oxidizing conditions that promote reactivity between the amino group and reducing sugar [24]. However, the brown intensity of the conventionally heated sample (3 h heating sample) was much higher than that of the ultrasound-assisted sample with a similar DG. Previous research reported that ultrasound can impede melanoidin formation by suppressing the polymerization of intermediate compounds [25]. In our results, we compared only conventional and ultrasound-assisted heating under specific conditions. Therefore, to understand the production of intermediate or end products in the Maillard reaction between BGS protein and GA, further research with varying kinetic parameters, including concentrations, mixture ratios, temperature, and so on, would be needed.

#### 3.1.3 Protein solubility

**Fig. 2:**
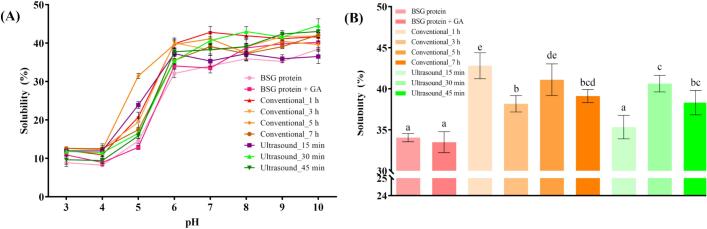
(A) Protein solubility at pH 3–10. (B) Protein solubility (%) of each MRPs at pH = 7.

Protein solubility across the pH range of 3–10 is presented in Fig. 2A. The formation of protein-polysaccharide conjugates during the initial stage of the Maillard reaction can significantly enhance protein solubility, as the introduction of hydrophilic groups enhances water interaction and the reduction of hydrophobic interactions prevents aggregation [26]. The lowest solubility values were observed at pH 4 for all samples, exemplified by the control, BSG protein, sample with a solubility value of 8.22 ± 0.21% (w/w). This trend is likely attributable to the proximity of this pH to the estimated isoelectric point (pI) of BSG protein, approximately 3.8 [27]. At the pI, increased hydrophobic interactions between electrically neutralized proteins promote aggregation, leading to minimal solubility. Conversely, solubility increased as the pH deviated from 3.8. The BSG protein + GA sample exhibited either no significant difference or a higher solubility value compared to the protein sample across the entire pH range. For canola protein, mixing with GA enhanced solubility due to the presence of more hydrophilic groups in the mixture compared to the protein alone [20]. This suggests that the interaction between BSG protein and GA forms a complex with enhanced hydrophilicity. Samples subjected to the Maillard reaction generally exhibited higher solubility than the BSG protein and BSG protein + GA samples. This relative improvement was particularly evident at pH 7, a mild condition, as illustrated in Fig. 2B. The BSG protein sample showed a solubility of 34.06 ± 0.51% (w/w), whereas the BSG protein + GA sample showed a solubility of 33.50 ± 1.28% (w/w). Although the difference was not statistically significant, the ultrasound_15 min sample exhibited a higher solubility value of 35.34 ± 1.43% (w/w). Samples subjected to conventional heating showed an initial increase in solubility after the Maillard reaction, followed by a subsequent decrease. A similar trend was reported by Zhang, Herneke, Langton, Johansson and Corredig [28], who explained that heat treatment induces protein unfolding and exposure of buried hydrophobic residues, thereby promoting extensive aggregation. As heating progresses, these hydrophobic interactions lead to the formation of large insoluble aggregates, which ultimately decrease the soluble protein fraction and reduce overall solubility. Ultrasound treatment is well known to enhance protein solubility, which might be associated with protein structure, free SH groups, S–S contents, and protein hydrophobicity [29]. However, our results showed the opposite, as samples subjected to ultrasound-assisted heating also exhibited an initial increase in solubility compared to the BSG protein, followed by a decrease with extended heating times. The initial increase in solubility is attributed to increased protein steric hindrance resulting from protein-polysaccharide binding, which prevents protein aggregation. However, continued sonication can lead to excessive oxidation of protein molecules, forming dimers or precipitates, which can lead to a decrease in solubility [30]. Therefore, to understand the reason ultrasound-assisted samples exhibit lower protein solubility in the results, further studies including S–S contents, free SH groups, and protein hydrophobicity would be needed.

#### 3.1.4 Protein secondary structures of MRPs

**Fig. 3:**
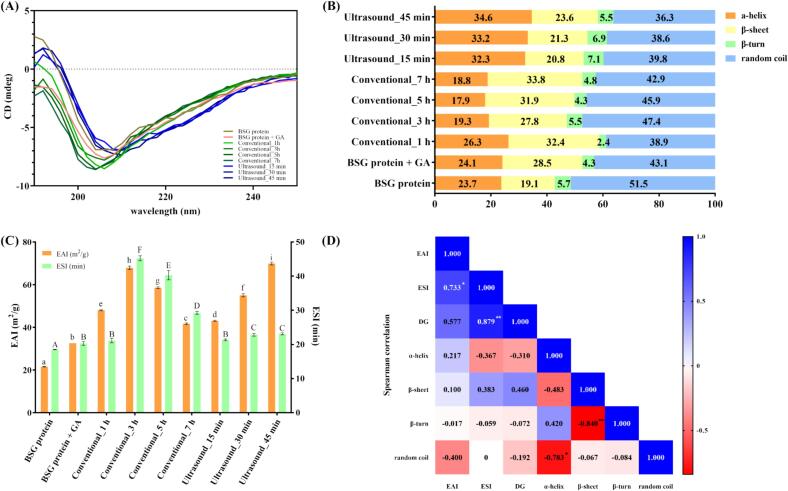
(A) Circular Dichroisms spectra. (B) Percentages of protein secondary structures (ɑ-helix, ß-sheet, ß-turn, and random coil). (C) EAI and ESI. (D) Spearman Correlation analysis between EAI, ESI, and protein secondary structures.

The CD spectrum and secondary structure of each sample are presented in Fig. 3A and B, respectively. The addition of GA to the BSG protein solution resulted in an increase in β-sheet content from 19.1% to 28.5%, while the random coil content decreased from 51.5% to 43.1%. A similar effect was observed when soy protein isolate (SPI) was mixed with flaxseed gum, leading to an increase in β-sheet content and a reduction in random coil content [31]. The authors attributed this change to the flaxseed gum’s ability to reduce the hydrogen bonds of SPI, which strengthened intermolecular interactions, resulting in an increase in β-sheet content and a decrease in random coil content.

The samples produced through conventional heating exhibited different trends compared to the BSG protein + GA sample. In the conventional_1 h sample, the α-helix content (26.3%) and β-sheet content (32.4%) increased, whereas the random coil content (38.9%) decreased. However, as heating continued, the α-helix content remained lower than that of the control, and the β-sheet content showed an initial decrease, followed by a gradual increase compared to conventional_1 h sample. The random coil content showed a similar trend, with an initial increase followed by a gradual decrease compared to conventional_1 h sample. These observations suggest that, initially, heating increases α-helix and β-sheet content, leading to a more ordered structure. However, in conventional_3 h sample, the α-helix content decreased due to glycation with polysaccharides. The decrease in α-helix content from conventional_3 h can be explained by research examining the reaction between soy protein hydrolysate (SPH) and GA. After the Maillard reaction, the α-helix content of the SPH-GA conjugate decreased, which was attributed to the formation of covalent bonds between the protein and GA, causing the screwed structure to unwind. The increase in β-sheet content with conventional heating can be attributed to the formation of covalent bonds during the Maillard reaction. The Maillard reaction reduces hydrogen bonds and enhances intermolecular interactions, resulting in an increase in β-sheet content [32].

The samples produced through ultrasound-assisted heating exhibited a 44% increase in α-helix content at 45 min compared to the BSG protein + GA mixture, accompanied by a decrease in β-sheet, β-turn, and random coil content. A study on sonication of β-lactoglobulin reported that α-helix content increased while β-sheet and β-turn content decreased. This change was attributed to the disruption of intermolecular interactions caused by sonication, leading to a reduction in β-sheet content and an increase in α-helix content [33]. These findings are consistent with the well-known mechanism in which ultrasound-induced cavitation disrupts hydrogen bonding and unfolds protein molecules. This unfolding loosens the tertiary structure and promotes the reorganization of secondary elements, where unstable random coil regions collapse and rearrange into more ordered α-helix structures. As a result, previously buried sites become exposed and the protein adopts a more defined global conformation [34]. The strong negative correlation between α-helix and random coil content (r = −0.783), as shown in Fig. 3D, further supports this structural shift. This trend is consistent with previous findings on the interaction between ultrasound and protein–sugar systems. One study showed that low-power ultrasonication (200 W for 30 min) reduced random coil structures while increasing β-sheet content in soybean protein [35]. Several researchers have reported that the shear and pressure generated by ultrasound also promote reactions between proteins and the aqueous phase, leading to backbone modifications. As these noncovalent interactions weaken, proteins become more unfolded and prone to aggregation, resulting in a more flexible and disordered structure that improves interfacial properties [36]. Moreover, ultrasonic cavitation can generate reactive species such as H∙ and ∙OH radicals, which can oxidize protein sulfhydryl groups and disrupt native structures. This structural disruption exposes additional amino groups, increasing their accessibility to reducing sugars and thereby facilitating the glycation reaction [36]. In addition, previous studies have shown that ultrasound-induced structural modifications can enhance the nutritional value of proteins by disrupting structural barriers, improving enzyme accessibility, and increasing the digestibility and bioavailability of essential amino acids. Such improvements in nutrient absorption and metabolic utilization highlight the positive nutritional implications of ultrasound-assisted protein modification [37]. These findings were further confirmed in a study where the Maillard reaction was induced between β-lactoglobulin and hyaluronic acid using ultrasound-assisted heating [38]. However, mathematical relationship between the observed structural modifications and the improvements in emulsifying performance will require further investigation.

### 3.2 Emulsifying properties of MRPs

#### 3.2.1 Emulsion activity index (EAI) and emulsion stability index (ESI)

The EAI and ESI values of MRPs prepared using BSG protein and GA are presented in Fig. 3C. The BSG protein sample exhibited an EAI of 21.6 ± 0.2 m^2^/g and an ESI of 18.5 ± 0.1 min, whereas the BSG protein + GA sample showed an EAI of 32.6 ± 0.0 m^2^/g and an ESI of 20.3 ± 0.5 min. Both indices increased with the addition of GA compared to the presence of BSG protein alone. A study examining the mixing and conjugation of quinoa protein with gum arabic reported similar enhancements in EAI and ESI [39]. The observed increase in EAI and ESI values in that study was attributed to improved amphiphilic and hydrophilic properties arising from the interaction between GA and the protein. It is plausible that a similar mechanism is responsible for the observed increase in EAI and ESI in the BSG protein + GA sample in the present study.

Samples subjected to conventional heating exhibited higher EAI and ESI values than the BSG protein, the control. Notably, the conventional_3 h sample showed a 108% increase in EAI and a 123% increase in ESI compared to the BSG protein + GA sample. Although the EAI and ESI values gradually decreased with extended heating up to 7 h, they remained higher than those of the control. The Maillard reaction between proteins and polysaccharides enhances steric hindrance around proteins due to the covalent attachment of polysaccharides. These conjugated proteins form a macromolecular film around emulsion droplets during emulsification, which increases steric hindrance, thereby preventing aggregation between the emulsion droplets and maintaining the emulsion structure [40]. In the heated sample, the α-helix decreased, and the β-sheet increased, leading to EAI, and ESI enhancement. A reduction in the α-helix (correlation with EAI: r = 0.217) leads to protein unfolding, exposing the protein's internal hydrophobic groups and thereby enhancing molecular flexibility. Molecular flexibility enhancement at the oil–water interface redistributes the oil–water interface and improves emulsifying activity [22], [41]. Also, the β-sheet is known to expose the protein's hydrophilic groups at the oil/water interface, thereby stabilizing the interface [11], which matches with the β-sheet to ESI correlation (r = 0.383). However, despite a reduction in a-helix and an increase in β-sheet, EAI and ESI decreased after 5 and 7 h of conventional heating can be attributed to a disruption in the balance between hydrophobicity and hydrophilicity. Generally, EAI and ESI exhibit an initial increase followed by a decline with prolonged heating. This trend arises because amphiphilic proteins stabilize the interface by interacting with both the oil and aqueous phases in a balanced manner [42]. Therefore, it can be inferred that the amphiphilic regions of BSG protein and GA were optimally oriented at 3 h of conventional heating, but this orientation deteriorated with extended heating. Additionally, prolonged heating may lead to the degradation of the initially formed MRPs, which reduces emulsifying properties [43].

Samples prepared using ultrasound-assisted heating also showed a trend of increasing EAI and ESI, similar to those produced through conventional heating. Particularly, the increase in EAI was more pronounced than the increase in ESI. The ultrasound_45 min sample showed a 115% increase in EAI and a 13% increase in ESI compared to the protein + GA sample, indicating a greater increase in EAI compared to ESI. Notably, the EAI of the ultrasound_45 min sample was comparable to that of the conventional_3 h sample, despite the latter involving approximately twice the heating time. The more rapid enhancement in emulsifying properties with ultrasound treatment can be attributed to sonication-induced protein unfolding, which exposes additional amino groups, thereby accelerating glycation and more rapidly enhancing emulsifying properties [44].

The enhancement in emulsifying properties achieved through the Maillard reaction can also be attributed to improvements in solubility. Both conventional and ultrasound-assisted heating increased the solubility of the samples across the entire pH range, enabling greater solubilization in water and consequently improving emulsifying properties. In addition to improved solubility, protein flexibility allows proteins to function more effectively at the interface [45]. Moreover, an increase in random coil content can lead to greater flexibility [46]. This suggests that the relatively higher random coil content, indicative of a more disordered structure, observed in the conventional_3 h sample enhanced its flexibility, resulting in the highest emulsifying properties among the conventionally heated samples. In contrast, samples subjected to ultrasound-assisted heating exhibited a relatively high α-helix content. Given that the α-helix structure is the most favorable for stabilizing the oil/water interface, the increased α-helix content in these samples likely accounts for their enhanced emulsifying properties [47]. The increase in α-helix content enhances the ability of the protein to rapidly adsorb and form stable interfacial films, which directly explains the improved emulsifying performance observed in our ultrasound-assisted heating samples [48]. Although the compositional analysis of the BSG protein fraction used in this study was not performed, previous reports indicate that alkaline extraction followed by acid precipitation does not yield a pure protein isolate. Instead, such fractions typically contain extracted non-protein constituents, including cell-wall polysaccharides (e.g., hemicellulose and arabinoxylan), insoluble and soluble dietary fibers, small amounts of lipids, and both bound and free phenolic compounds [49]. These components may interact with GA through hydrogen bonding, hydrophobic association, or polymer–polymer entanglement, thereby providing minor contributions to continuous-phase viscosity or interfacial stabilization [50]. However, their influence on the improvements in EAI and ESI is likely limited compared with the dominant effects arising from BSG protein + GA Maillard conjugation and ultrasound-induced structural modifications.

#### 3.2.2 Dynamic light scattering (DLS) analysis

**Fig. 4:**
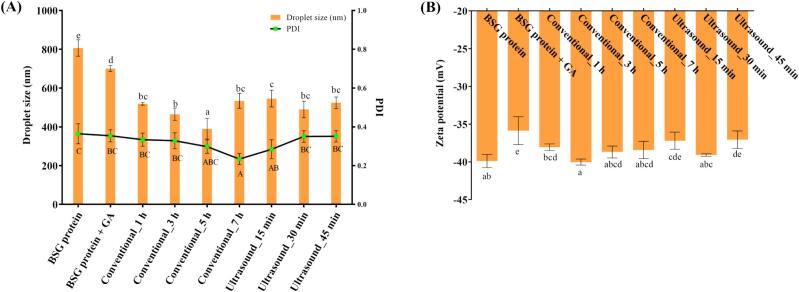
(A) Emulsion droplet size (column) and polydispersity index (PDI; line graph) of each emulsion samples. (B) Zeta potential (mV) values of each emulsion samples.

The z-average of the emulsions prepared using MRPs formed from BSG protein and GA is presented in Fig. 4A. Generally, smaller emulsion droplet sizes indicate superior emulsion capability [51]. The BSG protein + GA sample exhibited a droplet size approximately 87% that of the protein-only sample, a trend comparable to the EAI and ESI results. Consistent with the results of the EAI, the droplet size of the conventionally heated samples initially decreased and then increased with extended heating time. In contrast, the droplet size of the ultrasound-assisted heated samples decreased, with no statistically significant differences observed from the ultrasound_30 min sample. The PDI, which reflects the degree of droplet size distribution, is a key factor influencing emulsion stability [52]. Among the tested samples, the BSG protein exhibited the highest PDI (0.364 ± 0.052), whereas the conventional_7 h sample displayed the lowest value (0.234 ± 0.029). The PDI values of the emulsion samples showed trends similar to those observed for ESI. The emulsion properties can be influenced to a certain extent by the presence or absence of sonication during the emulsion preparation process. The difference from EAI and ESI can likely be explained by these methodological differences [53].

The zeta potential values of the emulsion droplets are presented in Fig. 4B. The absolute zeta potential value is indicative of the electrostatic repulsion of the emulsion, with absolute values exceeding 30 mV signifying a stable state [7]. The BSG protein + GA sample exhibited the lowest absolute value at −35.9 ± 1.9 mV, indicating that all emulsions prepared using the samples were electrically stable. Compared to the BSG protein sample, the BSG protein + GA emulsion exhibited a lower absolute value. A previous study reported a decrease in absolute zeta potential values when GA was added to zein and the Maillard reaction was carried out, compared to zein alone [7]. They attributed this to the lower charge of GA compared to the BSG protein, resulting in a smaller absolute value when the two were combined. Similarly, in this study, the high negative charge of BSG protein appeared to contribute to a reduction in the absolute zeta potential value when combined with GA. As the Maillard reaction progressed, the absolute values of the conventional_3 h (−40.0 ± 0.4 mV) and ultrasound_30 min (−39.1 ± 0.2 mV) samples increased, similar to the value of the protein sample (−39.9 ± 0.9 mV). This increase can be attributed to the decrease in the number of ionized amine groups of the protein as a result of their reaction with carbonyl groups during heating, leading to a reduction in the overall negative charge of the protein [54]. This improved electrostatic repulsion contributed to enhanced emulsion properties in MRPs produced through conventional heating and ultrasound-assisted heating.

#### 3.2.3 Inverted fluorescence microscopy

**Fig. 5:**
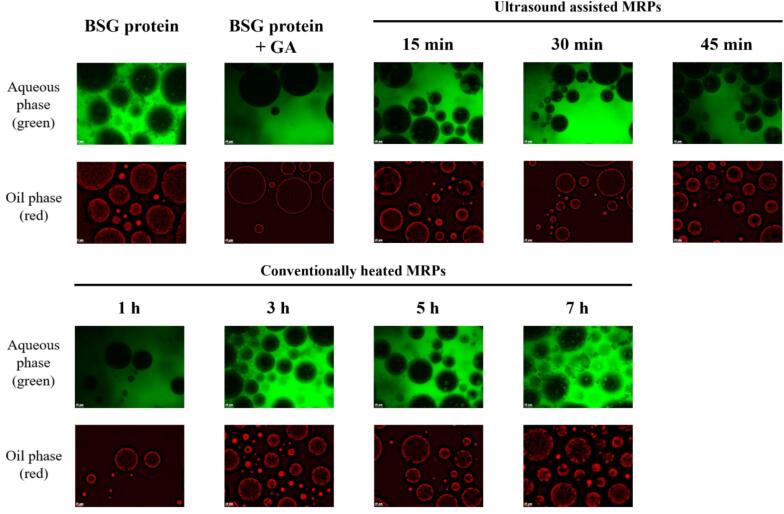
Fluorescence micrographs (40×) of each emulsion samples (aqueous phase: green, oil phase: red).

Fluorescence microscopy was employed to examine the microstructure of emulsions stabilized by MRPs derived from BSG protein and GA. FITC was used to stain the aqueous phase (green), and Nile Red was used to stain the oil phase (red). The resulting images are presented in Fig. 5. All samples exhibited an oil-in-water (O/W) emulsion morphology, with the aqueous phase (FITC-stained) enveloping the oil droplets (Nile Red-stained). Smaller, more dispersed emulsion droplets are indicative of superior emulsion properties [55]. Emulsions formed with samples subjected to the Maillard reaction displayed smaller and more dispersed droplets compared to the control. Consistent with the findings from the EAI, ESI, and DLS analyses results, the conventional_3 h and ultrasound_45 min samples demonstrated a more favorable emulsion structure, characterized by smaller and well-separated droplets.

### 3.3 Volatile compounds in MRPs

As previously reported, sonication enhanced flavor and reduced off-flavors. Due to the nature of BSG as a raw material, its off-flavor can influence the sample during extract preparation [17]. This could potentially affect the flavor of the emulsion. To determine the effect of sonication on MRP formation and off-flavor reduction, we analyzed the volatile compound composition.

To investigate the changes in flavor profiles, HS-SPME-GC–MS analysis was performed on samples subjected to varying conditions and durations of the Maillard reaction. The detected volatile compounds were classified into several groups, including esters, hydrocarbons, furan, alcohol, acids, aldehydes, and ketones (Table 1). The number and composition of volatile compounds varied significantly depending on the sample composition, Maillard reaction method, and reaction time. BSG protein and BSG protein + GA exhibited 12 and 18 volatiles, respectively. Conventional heating significantly increased the number of volatiles, with counts ranging from 38 (conventional_1 h) to 46 (conventional_7 h). In contrast, all samples subjected to ultrasound-assisted heating consistently showed only 10 types of volatile compounds.

**Table 1:** Peak area ratio (PAR) of volatile compounds in control group and Maillard reaction group.

No.	Compounds	Retention Index		Identification method	RT (min)	BSG protein	Gum Arabic							
		RI	RI (Ref.)				BSG protein + GA	Conventional_1 h	Conventional_3 h	Conventional_5 h	Conventional_7 h	Ultrasound_15 min	Ultrasound_30 min	Ultrasound_45 min
*Acids*														
1	Octanoic acid	2077	2075	MS, RI	62.718	ND	ND	ND	0.006 ± 0.010^a^	0.006 ± 0.011^a^	0.009 ± 0.008^a^	ND	ND	ND
2	Nonanoic acid	2184	2174	MS, RI	67.382	ND	ND	0.022 ± 0.003^a^	0.024 ± 0.003^a^	0.024 ± 0.002^a^	0.026 ± 0.003^a^	ND	ND	ND
Total Acids						ND	ND	0.022 ± 0.003^a^	0.029 ± 0.010^ab^	0.030 ± 0.011^ab^	0.035 ± 0.008^b^	ND	ND	ND

*Alcohols*														
3	1-Pentanol	1268	1260	MS, RI	36.387	ND	ND	0.006 ± 0.010^a^	0.029 ± 0.001^b^	0.040 ± 0.002^c^	0.050 ± 0.002^d^	ND	ND	ND
4	1-Hexanol	1370	1373	MS, RI	31.275	ND	0.113 ± 0.028^b^	0.034 ± 0.006^c^	0.041 ± 0.004^c^	0.043 ± 0.006^c^	0.031 ± 0.003^c^	ND	ND	ND
5	1-Octen-3-ol	1464	1460	MS, RI	35.552	0.322 ± 0.019^e^	0.246 ± 0.017^bc^	0.199 ± 0.021^a^	0.265 ± 0.020^cd^	0.263 ± 0.020^cd^	0.321 ± 0.009^e^	0.171 ± 0.008^cd^	0.208 ± 0.030^ab^	0.294 ± 0.049^d^
6	2-Heptanol	1472	1476	MS, RI	35.899	ND	ND	ND	ND	0.012 ± 0.011^a^	0.014 ± 0.012^a^	ND	ND	ND
7	2-Ethyl-1-hexanol	1504	1503	MS, RI	37.352	0.378 ± 0.012^d^	0.337 ± 0.009^c^	0.323 ± 0.009^c^	0.326 ± 0.013^c^	0.333 ± 0.011^c^	0.305 ± 0.003^b^	0.077 ± 0.009^c^	0.076 ± 0.002^a^	0.078 ± 0.011^a^
8	(E)-2-Hepten-1-ol	1529	1536	MS, RI	38.362	ND	0.026 ± 0.024^a^	ND	ND	ND	ND	ND	ND	ND
9	1-Octanol	1575	1573	MS, RI	40.288	ND	ND	0.035 ± 0.003^a^	0.045 ± 0.002^b^	0.052 ± 0.003^c^	0.058 ± 0.002^d^	ND	ND	ND
10	(Z)-2-Octen-1-ol	1634	1639	MS, RI	42.721	0.151 ± 0.001^c^	0.098 ± 0.01^ab^	0.093 ± 0.013^a^	0.113 ± 0.002^b^	0.094 ± 0.004^a^	0.108 ± 0.002^ab^	0.128 ± 0.006^b^	0.094 ± 0.008^a^	0.135 ± 0.023^c^
11	3-Cyclohexene-1-methanol	1723	1696	MS, RI	46.263	ND	ND	0.022 ± 0.001^a^	0.015 ± 0.013^a^	0.024 ± 0.005^a^	0.025 ± 0.004^a^	ND	ND	ND
Total Alcohols						0.849 ± 0.015^de^	0.818 ± 0.070^d^	0.709 ± 0.041^c^	0.832 ± 0.035^de^	0.858 ± 0.046^de^	0.909 ± 0.021^d^	0.374 ± 0.010^a^	0.377 ± 0.040^a^	0.506 ± 0.082^b^

*Aldehydes*														
12	Hexanal	1101	1110	MS, RI	17.897	0.356 ± 0.309^a^	0.163 ± 0.011^a^	1.291 ± 0.058^b^	1.760 ± 0.065^c^	2.137 ± 0.052^d^	2.424 ± 0.082^e^	0.221 ± 0.003^c^	0.216 ± 0.007^a^	0.283 ± 0.049^a^
13	(E)-2-Hexenal	1240	1240	MS, RI	24.965	ND	ND	0.008 ± 0.014^a^	0.014 ± 0.025^a^	0.040 ± 0.006^b^	0.040 ± 0.004^b^	ND	ND	ND
14	Octanal	1305	1306	MS, RI	28.293	ND	ND	0.058 ± 0.003^a^	0.084 ± 0.004^b^	0.111 ± 0.016^c^	0.116 ± 0.007^c^	ND	ND	ND
15	(E)-2-Heptenal	1346	1342	MS, RI	30.161	0.394 ± 0.016^e^	0.078 ± 0.012^a^	ND	0.225 ± 0.012^c^	0.164 ± 0.008^b^	0.258 ± 0.021^c^	0.265 ± 0.018^c^	0.303 ± 0.015^d^	0.453 ± 0.061^f^
16	Nonanal	1411	1410	MS, RI	33.219	ND	ND	0.120 ± 0.013^a^	0.109 ± 0.094^a^	0.225 ± 0.032^b^	0.201 ± 0.040^b^	ND	ND	ND
17	(E)-2-Octenal	1453	1463	MS, RI	35.049	0.238 ± 0.014^e^	0.081 ± 0.015^a^	0.122 ± 0.009^bc^	0.131 ± 0.008^bcd^	0.115 ± 0.005^b^	0.145 ± 0.011^d^	0.125 ± 0.008^bcd^	0.136 ± 0.006^cd^	ND
18	(E,E)-2,4-Heptadienal	1518	1515	MS, RI	37.936	ND	ND	0.052 ± 0.017^a^	0.093 ± 0.003^b^	0.114 ± 0.004^c^	0.086 ± 0.007^b^	ND	ND	ND
19	Benzaldehyde	1554	1557	MS, RI	39.415	0.110 ± 0.005^b^	0.021 ± 0.019^a^	0.380 ± 0.007^c^	0.659 ± 0.026^d^	0.879 ± 0.030^e^	1.009 ± 0.049^f^	0.093 ± 0.003^d^	0.085 ± 0.004^b^	0.084 ± 0.008^b^
20	(E)-2-Nonenal	1560	1569	MS, RI	39.683	ND	ND	0.042 ± 0.005^a^	0.052 ± 0.004^b^	0.055 ± 0.004^b^	0.055 ± 0.006^b^	ND	ND	ND
21	2-Butyl-2-octenal	1690	1688	MS, RI	44.994	0.021 ± 0.036^ab^	0.041 ± 0.004^b^	0.048 ± 0.042^b^	0.090 ± 0.018^c^	0.083 ± 0.007^c^	0.088 ± 0.012^c^	ND	ND	ND
22	(E,E)-2,4-Nonadienal	1729	1741	MS, RI	46.518	ND	ND	0.043 ± 0.005^a^	0.052 ± 0.005^b^	0.057 ± 0.003^b^	0.057 ± 0.007^b^	ND	ND	ND
23	4-Ethylbenzaldehyde	1742	1745	MS, RI	40.007	ND	ND	ND	0.009 ± 0.016^ab^	0.023 ± 0.020^bc^	0.037 ± 0.003^c^	ND	ND	ND
24	(E,E)-2,4-Decadienal	1837	1848	MS, RI	51.099	ND	ND	ND	0.007 ± 0.012^a^	0.028 ± 0.002^b^	0.030 ± 0.003^b^	ND	ND	ND
25	4-Propylbenzaldehyde	1862		MS	52.316	ND	ND	0.034 ± 0.020^a^	0.061 ± 0.002^b^	0.065 ± 0.005^b^	0.070 ± 0.003^b^	0.180 ± 0.010^b^	0.180 ± 0.010^cd^	0.203 ± 0.011^d^
Total Aldehydes						1.342 ± 0.276^c^	0.382 ± 0.038^a^	2.192 ± 0.093^d^	3.340 ± 0.222^e^	4.087 ± 0.070^f^	4.608 ± 0.156^g^	0.882 ± 0.030^b^	0.922 ± 0.031^b^	1.022 ± 0.127^b^

*Esters*														
26	Hexyl hexanoate	1626	1619	MS, RI	42.409	ND	ND	0.011 ± 0.010^ab^	0.006 ± 0.009^ab^	0.015 ± 0.013^bc^	0.024 ± 0.004^c^	ND	ND	ND
27	Methyl salicylate	1812	1813	MS, RI	49.934	7.824 ± 0.277^e^	0.238 ± 0.047^b^	0.098 ± 0.012^ab^	0.053 ± 0.002^ab^	0.017 ± 0.015^ab^	ND	5.508 ± 0.206^ab^	5.079 ± 0.127^c^	5.448 ± 0.194^d^
Total Esters						7.824 ± 0.277^d^	0.238 ± 0.047^a^	0.109 ± 0.017^a^	0.058 ± 0.008^a^	0.031 ± 0.027^a^	0.024 ± 0.004^a^	5.508 ± 0.206^c^	5.079 ± 0.127^b^	5.448 ± 0.194^c^

*Furans*														
28	2-Pentylfuran	1247	1230	MS, RI	25.33	ND	ND	0.053 ± 0.002^a^	0.054 ± 0.005^a^	0.054 ± 0.005^a^	0.058 ± 0.004^a^	ND	ND	ND
29	Furfural	1485	1489	MS, RI	36.485	ND	ND	0.032 ± 0.002^a^	0.040 ± 0.003^ab^	0.049 ± 0.002^b^	0.043 ± 0.018^b^	ND	ND	ND
Total Furans						ND	ND	0.084 ± 0.003^a^	0.093 ± 0.007^ab^	0.102 ± 0.006^b^	0.101 ± 0.014^b^	ND	ND	ND

*Hydrocarbons*														
30	Tetradecane	1400	1400	MS, RI	32.744	ND	ND	0.029 ± 0.014^ab^	0.031 ± 0.002^ab^	0.038 ± 0.005^b^	0.027 ± 0.006^a^	ND	ND	ND
31	3-Ethyl-2-methyl-1,3-hexadiene	1446	1421	MS, RI	34.727	ND	ND	0.063 ± 0.006^a^	ND	ND	ND	ND	ND	ND
32	Hexadecane	1600	1600	MS, RI	41.368	ND	ND	0.011 ± 0.018^a^	ND	ND	ND	ND	ND	ND
Total Hydrocarbons						ND	ND	0.102 ± 0.010^c^	0.031 ± 0.002^ab^	0.038 ± 0.005^b^	0.027 ± 0.006^a^	ND	ND	ND

*Ketones*														
33	2-Heptanone	1199	1199	MS, RI	23.057	ND	ND	0.280 ± 0.015^a^	0.435 ± 0.020^b^	0.570 ± 0.004^c^	0.655 ± 0.025^d^	ND	ND	ND
34	3-Octanone	1273	1244	MS, RI	26.632	ND	0.015 ± 0.022^a^	ND	ND	ND	ND	ND	ND	ND
35	2-Octanone	1301	1302	MS, RI	28.133	ND	0.055 ± 0.011^a^	0.091 ± 0.003^b^	0.176 ± 0.008^c^	0.258 ± 0.003^d^	0.312 ± 0.018^e^	ND	ND	ND
36	1-Octen-3-one	1319	1315	MS, RI	28.927	0.136 ± 0.008^d^	ND	0.064 ± 0.005^a^	0.081 ± 0.008^abc^	0.074 ± 0.003^ab^	0.088 ± 0.006^bc^	0.080 ± 0.011^abc^	0.092 ± 0.011^cd^	0.145 ± 0.019^d^
37	2,3-Octanedione	1337	1335	MS, RI	29.741	ND	ND	0.034 ± 0.012^a^	0.040 ± 0.004^ab^	0.045 ± 0.011^b^	0.030 ± 0.005^a^	ND	ND	ND
38	6-Methyl-5-hepten-2-one	1357	1357	MS, RI	30.163	ND	ND	0.033 ± 0.002^a^	0.048 ± 0.003^b^	0.072 ± 0.013^c^	0.082 ± 0.001^d^	ND	ND	ND
39	2-Nonanone	1407	1405	MS, RI	33.067	ND	0.064 ± 0.003^a^	ND	0.116 ± 0.128^a^	0.038 ± 0.033^a^	0.058 ± 0.004^a^	ND	ND	ND
40	(E)-3-Octen-2-one	1430	1440	MS, RI	34.044	0.098 ± 0.004^b^	0.033 ± 0.001^a^	0.196 ± 0.008^c^	0.226 ± 0.010^d^	0.240 ± 0.006^e^	0.239 ± 0.010^e^	ND	ND	ND
41	2-Decanone	1512	1515	MS, RI	37.693	ND	ND	0.006 ± 0.010^a^	0.030 ± 0.002^b^	0.042 ± 0.004^c^	0.051 ± 0.003^d^	ND	ND	ND
42	3-Nonen-2-one	1536	1547	MS, RI	38.652	ND	ND	0.017 ± 0.015^a^	0.037 ± 0.006^b^	0.059 ± 0.003^c^	0.080 ± 0.006^d^	ND	ND	ND
43	(E,E)-3,5-Octadien-2-one	1544	1549	MS, RI	38.985	ND	ND	0.048 ± 0.002^a^	0.058 ± 0.004^c^	0.058 ± 0.003^c^	0.053 ± 0.004^b^	ND	ND	ND
44	2-Undecanone	1618	1615	MS, RI	42.112	ND	ND	ND	ND	0.019 ± 0.016^a^	0.032 ± 0.003^b^	ND	ND	ND
45	Acetophenone	1685	1690	MS, RI	44.992	ND	ND	ND	0.016 ± 0.028^ab^	0.052 ± 0.017^c^	0.034 ± 0.003^bc^	ND	ND	ND
46	Geranylacetone	1875	1877	MS, RI	52.988	ND	ND	ND	ND	0.013 ± 0.011^a^	0.023 ± 0.001^b^	ND	ND	ND
Total Ketones						0.233 ± 0.010^b^	0.166 ± 0.031^ab^	0.766 ± 0.037^c^	1.260 ± 0.115^de^	1.533 ± 0.057^e^	1.732 ± 0.063^f^	0.080 ± 0.011^a^	0.092 ± 0.011^a^	0.145 ± 0.019^ab^
**Others**														
47	Methoxy-phenyl-oxime	1755		MS	47.535	ND	ND	0.019 ± 0.016^ab^	0.010 ± 0.017^a^	0.033 ± 0.003^b^	0.035 ± 0.002^b^	ND	ND	ND
48	Guaiacol	1887	1880	MS, RI	53.603	ND	ND	ND	0.043 ± 0.004^a^	0.079 ± 0.010^b^	0.110 ± 0.009^c^	ND	ND	ND
Total Others						ND	ND	0.019 ± 0.016^a^	0.052 ± 0.015^b^	0.112 ± 0.011^c^	0.145 ± 0.008^d^	ND	ND	ND
Total						10.247 ± 0.501^g^	1.603 ± 0.032^a^	4.000 ± 0.146^b^	5.691 ± 0.298^c^	6.787 ± 0.075^de^	7.576 ± 0.183^f^	6.842 ± 0.252^de^	6.463 ± 0.169^d^	7.119 ± 0.412^ef^

Values were presented as mean ± standard deviation (n = 3). Different letters above each data in a row indicated significant differences (Duncan test, p < 0.05) for the same row. ND: not detected. Kovats retention indices were based on DB-WAX UI in NIST database. For the identification of each volatile component, MS analysis was performed by comparing mass spectra with the Wiley mass database, while RI analysis was conducted by comparing calculated Kovats retention index values using alkane standards with those reported in the literature.

The addition of GA to the BSG protein sample resulted in a distinct volatile profile compared to the BSG protein alone. Notably, the concentration of methyl salicylate decreased to 3.04% in the protein sample, and most existing volatiles exhibited reduced PAR values. Conversely, new volatiles, such as 1-hexanol, (E)-2-hepten-1-ol, 3-octanone, 2-octanone, and 2-nonanone, were detected, likely originating from the inclusion of GA. A similar phenomenon of reduced volatile levels was observed in pea protein hydrolysate mixed with GA, where decreased volatile concentrations were attributed to increased adsorption by the GA polypeptide backbone and entrapment within the GA structure [56].

Compared to BSG protein + GA sample, conventional heating uniquely generated volatiles belonging to acid, furan, and hydrocarbon groups, along with methoxy-phenyl-oxime compared to the BSG protein + GA sample. These compounds are likely products of Maillard reactions induced by heating. Alcohols such as 1-hexanol and 2-Ethyl-1-hexanol, which impart a sweet aroma to the emulsion, tended to decrease with heating, regardless of sonication. Notably, hexanal and benzaldehyde exhibited high PAR values that increased with prolonged heating, further substantiating their association with the Maillard reaction. However, the PAR values of hexanal and benzaldehyde were significantly lower in the sonicated sample than in the conventionally heated sample, suggesting that ultrasound may have a positive effect on reducing the content of potentially carcinogenic aldehydes such as hexanal [57]. Samples prepared using ultrasound-assisted heating displayed higher levels of methyl salicylate than BSG protein + GA sample. The lower number of volatile compounds observed under ultrasound-assisted Maillard reaction conditions can be attributed to physicochemical mechanisms rather than simple thermal vaporization. Ultrasound induces acoustic cavitation, generating micro-hot spots with transient high temperatures and pressures, as well as radical species, which collectively accelerate early Maillard pathways such as the Amadori rearrangement and α-dicarbonyl formation. This shift promotes the redistribution of reaction carbon flux toward nonvolatile melanoidins and protein–sugar crosslinked structures rather than low-molecular-weight volatile degradation products [23]. In addition, radicals produced during cavitation can facilitate oxidation, condensation, and polymerization of aldehydes and ketones, thereby suppressing their accumulation [58]. Cavitation-induced stripping and the entrapment of volatiles within the BSG protein–GA matrix may further contribute to the reduced detection of volatile compounds in ultrasound-treated samples [59]. Thus, this difference may be attributed to volatile loss during heating, as the open beaker setup allowed vaporization. Ultrasound treatment of samples in this open environment appears to be a methodological variable that influenced the volatilization of flavor components. Further research is needed to determine whether differences in volatile compound composition occur in sealed vials.

**Fig. 6:**
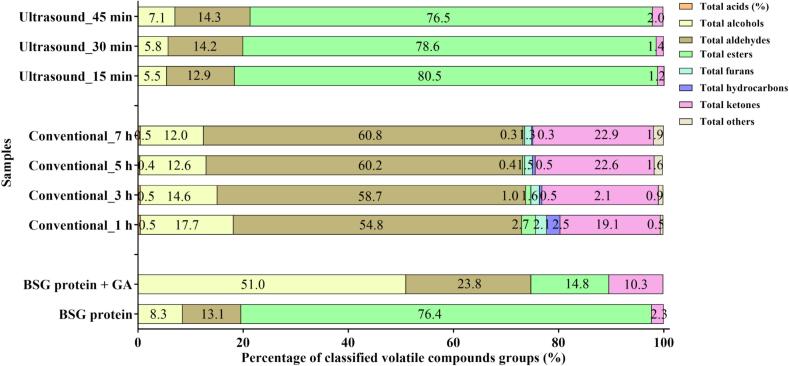
Percentage of classified volatile compound groups in control group and Maillard reaction group.

The distribution of volatile compound classes shifted in response to GA addition and the applied heating methods (Fig. 6). In the control protein sample, the addition of GA led to a reduction in ester content and increases in alcohols, aldehydes, and ketones. Conventional heating significantly increased the proportions of aldehydes and ketones while reducing alcohol content compared to BSG protein + GA. Ultrasound-assisted heating samples exhibited a similar trend, with increases in some alcohols and aldehydes. These observations are consistent with earlier findings that have reported an increase in aldehydes and ketones during Maillard reactions, highlighting their pivotal role in flavor profile development [60].

Unlike the conventional heating sample, (E)-2-hexenal, (E,E)-2,4-decadienal, nonanal, octanal, (E,E)-2,4-nonadienal, 2,3-octanedione, 2-undecanone, and 2-pentylfuran were not detected in the ultrasonic-treated sample. These compounds are known as unpleasant off-flavors in the form of aldehydes, ketones, and furans, which are closely related to the oxidation of fatty acids and lipids such as linoleic acid, and play a role in reducing the flavor of emulsifiers [61]. Except for hexanal and (E)-2-octenal, all of them were not detected in the BSG protein + GA sample and the ultrasonic-treated sample, but they were detected in the conventional heating sample. Furthermore, sonication inhibited the production of acids resulting from lipid oxidation, such as octanoic acid and nonanoic acid [62]. This suggests that major lipid components such as linoleic acid in brewer's grains are also contained in the BSG extract, and that their oxidation affected the flavor of the emulsifier. Ultrasonic treatment at a frequency of 20 kHz not only caused physical changes to the sample, but also generated local high-energy, high-temperature cavitation bubbles inside the sample, which may have generated free radicals and caused chemical structural changes. These free radicals are believed to have caused the proliferation of hydrophobic residues and protein oxidation in the BSG extract and GA proteins [58]. Structural modification through protein oxidation may have promoted the Maillard reaction. The absence of this unpleasant off-flavor indicates that ultrasound not only enhanced the emulsifying ability of the emulsifier in a short period of time without affecting lipid oxidation, but also promoted the production of MRPs, such as intermediate MRPs and melanoidin, which exhibit antioxidant properties, effectively improving flavor [63]. Additionally, ultrasonic treatment improved the biological safety of the resulting emulsions by inhibiting the formation of toxic volatile compounds such as α,β-unsaturated aldehydes, such as (E,E)-2,4-Decadienal and (E)-2-hexenal, which have the potential to cause genotoxicity and chronic inflammatory responses and cancer [64].

## 4 Conclusions

This study demonstrates that the Maillard reaction between BSG protein and GA significantly enhances the emulsifying properties of MRPs by improving solubility, altering secondary structures, and reducing emulsion droplet size. Furthermore, the presence of 2-pentylfuran and furfural in conventionally heated samples indicates that the Maillard reaction plays a role in modulating the flavor profile. These findings indicate that BSG protein, an abundant industrial by-product, can be upcycled into a plant-based natural emulsifier with improved functional properties. The enhanced EAI, ESI, and interfacial stability observed in this study further support the potential applicability of BSG protein + GA MRPs in clean-label emulsifiers, plant-based beverages, dressings and sauces, and nutraceutical delivery systems. Although the BSG protein extract may contain non-protein materials such as polysaccharides, lipids, and phenolics, these constituents are unlikely to have exerted a substantial influence on the emulsifying outcomes. Nevertheless, their minor contributions cannot be completely excluded, and future work will include compositional characterization to more precisely evaluate their functional role. In addition, future studies should include a safety evaluation of these modified protein systems to ensure their suitability for food applications. Nevertheless, ultrasound assisted Maillard reactions have potential economic benefits, as they can achieve comparable degrees of protein–sugar conjugation with substantially shorter reaction times and milder thermal conditions relative to conventional heating. These advantages suggest reduced energy input and improved processing efficiency. However, industrial implementation requires reactor systems capable of delivering uniform acoustic energy at high throughput. Continuous-flow ultrasonic reactors are considered more suitable for large scale operations than probe-based batch systems, but their performance depends on effective cavitation distribution and energy utilization. Differences in sonication methods may affect the performance of the produced emulsions. Moreover, optimization of protein-to-gum arabic ratios and total biopolymer concentrations will be needed to ensure cost-effectiveness and functional stability in real food systems. Therefore, pilot-scale evaluation using continuous-flow configurations is needed to verify process stability, energy efficiency, and overall scalability under realistic industrial conditions. Overall, these findings highlight the potential of the Maillard reaction as an effective strategy for enhancing the emulsifying and flavor properties of BSG protein.

## CRediT authorship contribution statement

**Mingyu Kim:** Writing – original draft, Formal analysis, Conceptualization. **Hyunwoo Ahn:** Methodology. **Yujin Wang:** Validation, Formal analysis. **Beomkyung Cho:** Validation, Methodology. **Woojin Na:** Methodology. **Kwang-Geun Lee:** Writing – original draft, Supervision, Investigation, Funding acquisition, Conceptualization.

## Declaration of competing interest

The authors declare that they have no known competing financial interests or personal relationships that could have appeared to influence the work reported in this paper.
